# Cyclopia with shoulder dystocia leading to an obstetric catastrophe: a case report

**DOI:** 10.1186/1752-1947-4-160

**Published:** 2010-05-27

**Authors:** Mahesh C Koregol, Mrutyunjaya B Bellad, Baburao R Nilgar, Mrityunjay C Metgud, Geeta Durdi

**Affiliations:** 1 Department of OBGYN, Dr BR Ambedkar Medical College, Rajiv Gandhi University of Health Sciences, Bangalore, Karnataka State, India; 2 Department of OBGYN, Jawaharlal Nehru Medical College, KLE University, Belgaum, Karnataka State, India

## Abstract

**Introduction:**

Cyclopia is a rare fetal malformation characterized by a single palpebral fissure and a proboscis associated with severe brain malformations. Approximately 1.05 in 100,000 births including stillbirths are identified as cyclopean. The prevalence is about one in 11,000 to 20,000 in live births and one in 250 during embryogenesis.

**Case presentation:**

A 30-year-old Indian woman of Asian origin, sixth gravida, was referred to the labor room of our hospital. There were no ultrasound examinations performed during this pregnancy as our patient had not received regular antenatal care. We found out that the head of her baby was already outside the vulva but the remaining parts of the baby were not yet delivered. Further examination was carried out and a diagnosis of shoulder dystocia with intrauterine fetal demise was made. A stillborn baby boy of 3.5 kg was delivered using McRoberts' maneuver. The baby was suspected of having features of cyclopia and this was later confirmed by autopsy and anatomic correlation. The mother had a cervical tear which extended into the lower segment of her uterus, thus leading to the rupture of her uterus. There was a massive broad ligament hematoma on the left side of her uterus. A total abdominal hysterectomy was carried out.

**Conclusion:**

Prenatal diagnosis by ultrasound examination might help in detecting cyclopia and preventing complications associated with this condition. However, in developing countries where women do not receive regular antenatal care and do not undergo prenatal diagnosis, such cases will go undetected. In our case report, the occurrence of shoulder dystocia could be coincidental, as no risk factors were previously noted.

## Introduction

Cyclopia is a serious median faciocerebral development deformity [1]. In most cases, craniofacial abnormalities are associated and the degree of severity is reflected in 80% of cases [2]. The severity has a marked variability and ranges from cyclopia to minimal craniofacial dysmorphism, such as mild microcephaly with a single central incisor [2].

Cyclopia is a rare fetal malformation characterized by a single palpebral fissure and a proboscis associated with severe brain malformations. Approximately 1.05 in 100,000 births, including stillbirths, are identified as cyclopian [3]. The prevalence of holoprosencephaly (HPE) is about one in 11,000 to 20,000 live births and one in 250 embryogenesis [2]. The etiology of holoprosencephaly is heterogeneous and comprises environmental factors such as maternal diabetes and other genetic causes. Cyclopia with HPE is an infrequent congenital anomaly of the forebrain system [1]. HPE results from incomplete cleaving of the telencephalic vesicles. True cyclopia is a rare anomaly in which the organogenetic development of the two separate eyes is suppressed [4]. Cyclopia is not compatible with life [3].

## Case presentation

A 30-year-old Indian woman of Asian origin, sixth gravida, was referred to our hospital's labor room from a private hospital as a case of obstructed labor. She was in her full-term pregnancy and experiencing spontaneous onset of labor. She had five living children who were delivered normally without any congenital malformations. All previous deliveries were normal and there was no history of shoulder dystocia. Our patient had not received any antenatal care during her sixth pregnancy. Since no ultrasound examinations were done on our patient, a diagnosis of congenital malformations could not have been made.

**Figure 1:**
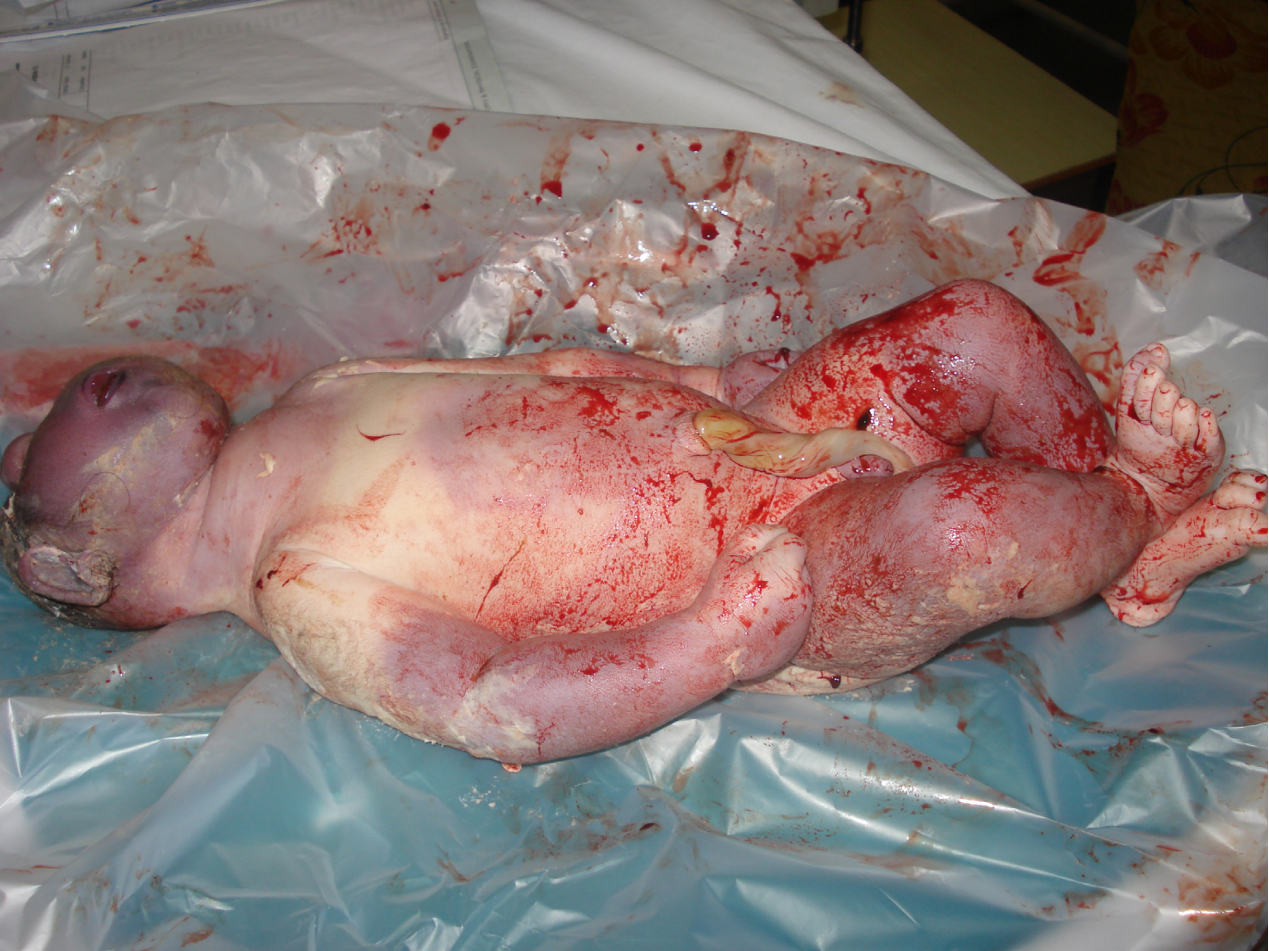
**Baby with cyclopia**.

On examination, our patient was well-built and nourished. She was five feet, two inches tall and weighed 74 kg. She appeared dehydrated and exhausted. Her uterus was contracting, but fetal heart sounds were not audible. We found out that the head of her baby was delivered outside her vulva but remaining parts of the baby were not delivered. Further examination was carried out and a diagnosis of unilateral shoulder dystocia with intrauterine fetal demise was made. The thighs of our patient were abducted, flexed on to her abdomen, and suprapubic pressure was given. A stillborn baby boy of 3.5 kg was delivered using McRoberts' maneuver (Figure 1).

**Figure 2:**
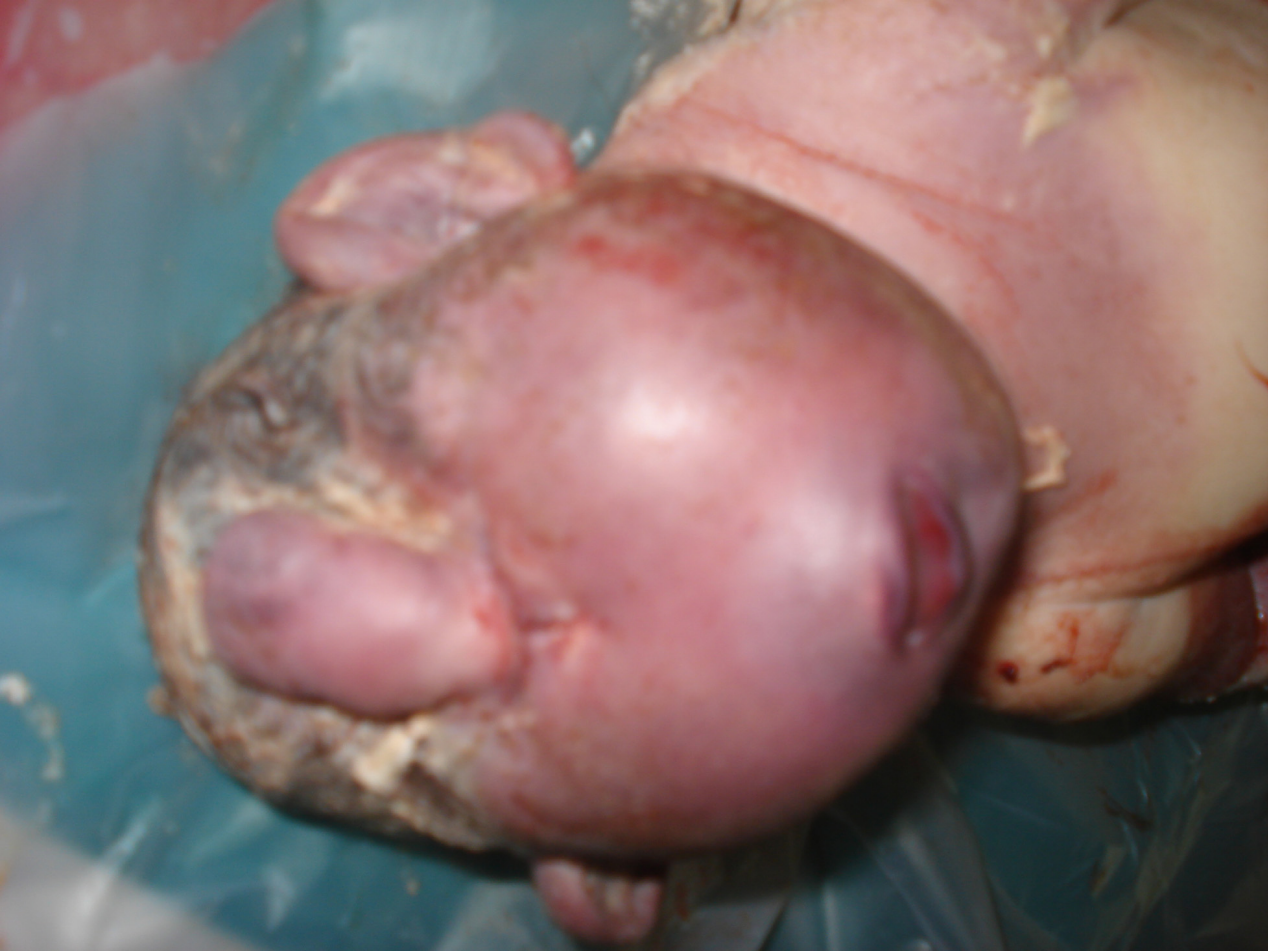
**Facial profile of the baby with cyclopia**.

The baby was noted to have a dysmorphic face with small mouth and chin (micrognathia). His nose could not be seen (Figure 2). A projection in the center of his forehead was suggestive of rudimentary eye with proboscis. The baby was suspected to have features of cyclopia and was sent to autopsy for further examination and anatomic correlation.

**Figure 3:**
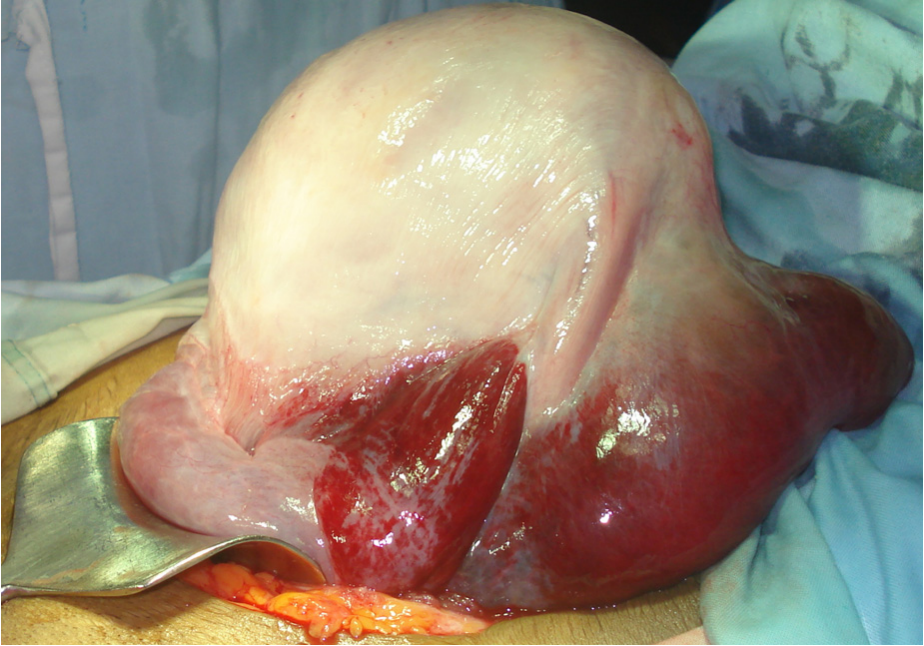
**Lateral view of the hematoma found at the mother's uterus**.

**Figure 4:**
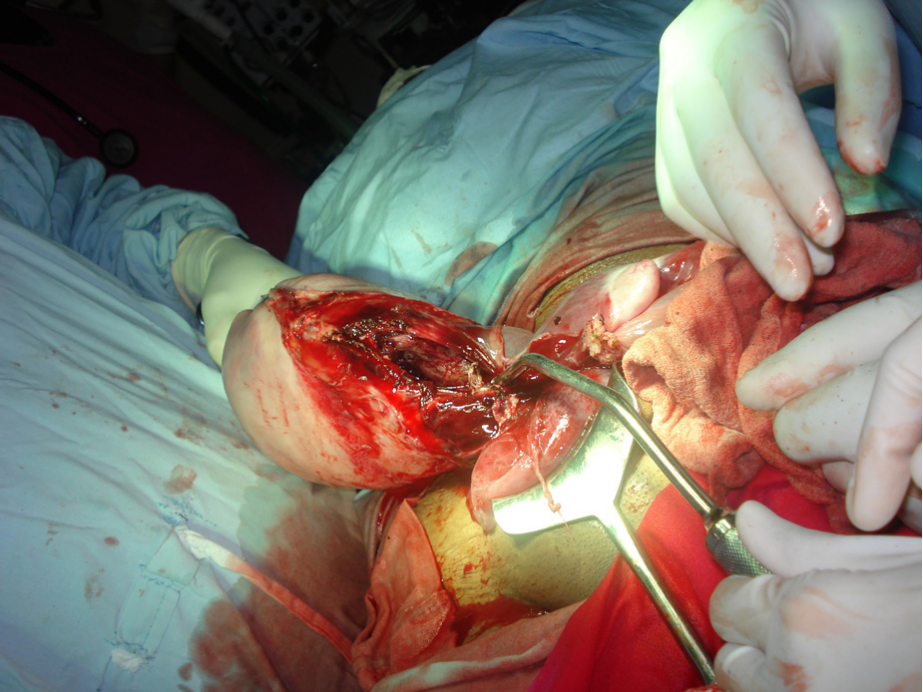
**Ruptured uterus of the mother**.

Our patient experienced a sudden gush of blood after she delivered her baby. An exploration of her cervix revealed a tear extending high up into her cervix. Our patient and her relatives were counselled about the possible extension of the cervical tear into the lower segment of her uterus and the necessity to perform laparotomy. An informed consent for laparotomy and hysterectomy was taken after explaining the risks involved. Laparotomy was immediately done under general anaesthesia and massive broad ligament hematoma (about 15 × 5 cm) was seen on the left side of her uterus (Figure 3). The hematoma was due to the extension of the cervical tear in to left lateral aspect of her uterus, thus leading to uterine rupture of about 10 cm in length (Figure 4). A total abdominal hysterectomy was subsequently done. Our patient was transfused with adequate amounts of blood products and other fluids throughout the procedure. Her post-operative period was uneventful.

**Figure 5:**
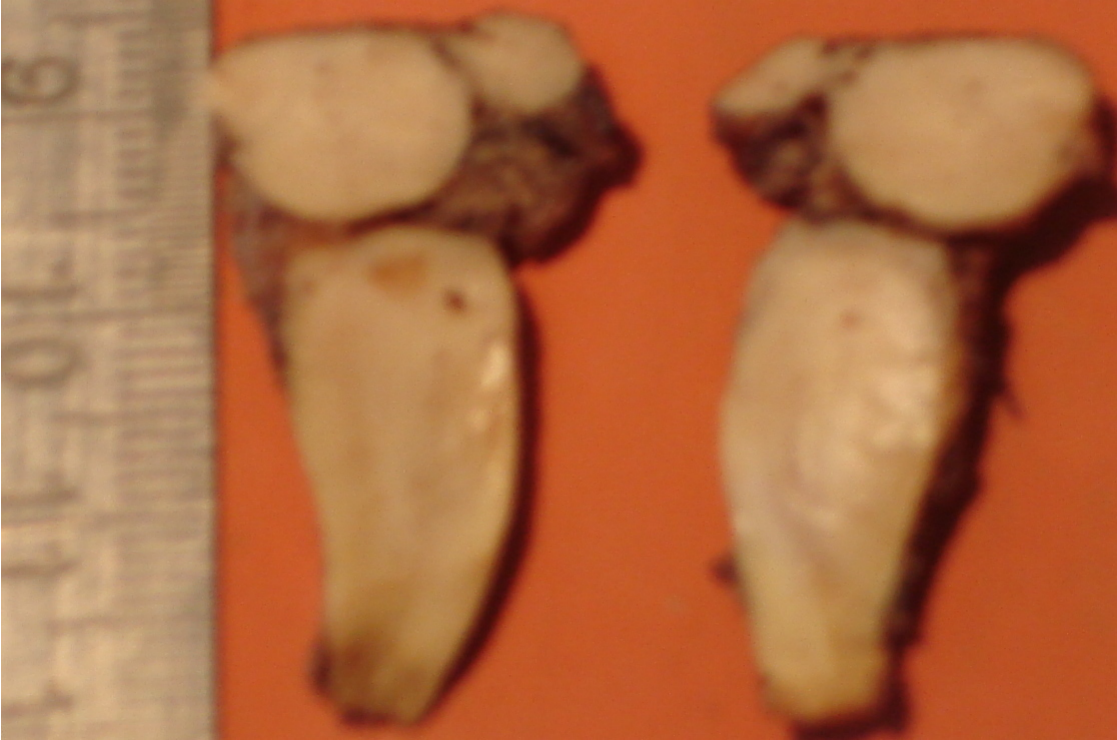
**Sections of the baby's brain (holoprosencephaly)**.

An autopsy of the baby confirmed the diagnosis of cyclopia (single centrally placed rudimentary eye along with proboscis). A dissection of the baby's brain showed hypoplastic holoprosencephaly (Figure 5). All other organs of the baby were normal and no other congenital malformations were found.

## Discussion

During embryogenesis, the prechordal mesoderm not only forms the median facial bones but also induces rostral neuroectodermal differentiation and morphogenesis [1]. Defects in the prechordal mesoderm due to mechanical, genetic or environmental teratogens can lead to the arrest or malformation of the facial bones, thus leading to micrognathia [1] as in our case. There are three types of eye deformities seen in cyclopia: one eye (monophthalmia), two fused eyeballs (synophthalmia) or complete absence of eyeballs (anophthalmia). The baby we describe in this case report had anophthalmia (Figure 2).

Approximately 50% of patients with HPE are associated with cytogenetic abnormality or monogenic syndrome [2]. Those who have extracephalic anomalies along with HPE are usually found to have trisomy 13 or 18 and triploidy [1]. However, exceptions to these observations exist [1]. In this case, no karyotype studies were carried out on the fetus due to the unavailability of resources.

To account for the evolution of cases with normal karyotype [5], maternal rubella, toxoplasmosis, alcoholism, diabetes, and drug treatment have been implicated as the etiological agents [1]. Few authors have described the role of maternal ingestion of drugs like salicylates in the evolution of cyclopia and other anomalies [1,6]. Our patient had no history of taking calculates or any other drugs during her pregnancy.

The 3D/4D sonograms helped our patient and her husband to visualize their baby's abnormalities and facilitated their acceptance of perinatal genetic counseling [7]. Various authors have observed that early diagnosis of this anomaly is possible by using the ultrasound examination, and the mother can be counseled in preparation for pregnancy termination [5,8,9]. Unfortunately, our patient was not registered for antenatal care in our hospital; hence no ultrasound examinations could be performed earlier. As such, a diagnosis of cyclopia could not be made during our patient's antenatal period.

The case we describe here is the first reported case of cyclopia that presented with shoulder dystocia during delivery. There were no particular high risk factors for shoulder dystocia in our patient, and it could in fact be a mere coincidence. The risk factor for ruptured uterus could be her parity as she was already on her sixth gravida.

## Conclusion

Prenatal diagnosis by ultrasound examination might help in the detection of cyclopia and in the prevention of complications associated with such a condition. However, in developing countries where women do not receive regular antenatal care and do not undergo prenatal diagnosis, such cases will go undetected. In the case we describe here, the occurrence of shoulder dystocia could be merely coincidental, as there were no high risk factors for the development of shoulder dystocia.

## Competing interests

The authors declare that they have no competing interests.

## Authors' contributions

MCK and MB were responsible for the concept of the case report. MCK wrote the manuscript. BN, MM and GD reviewed and edited the manuscript. All authors read and approved the final manuscript.

## Consent

Written informed consent was obtained from our patient for publication of this case report and any accompanying images. A copy of the written consent is available for review by the Editor-in-Chief of this journal.
